# The weekend effect: does hospital mortality differ by day of the week? A systematic review and meta-analysis

**DOI:** 10.1186/s12913-018-3688-3

**Published:** 2018-11-20

**Authors:** Kate Honeyford, Elizabeth Cecil, Michelle Lo, Alex Bottle, Paul Aylin

**Affiliations:** 1 Department of Primary Care and Public Health, Dr Foster Unit at Imperial College, 3 Dorset Rise, London, EC4Y 8EN UK; 2 Department of Family Medicine and Primary Healthcare, Hospital Authority, Hong Kong, Hong Kong

**Keywords:** Quality of care, Hospital, Weekend effect, Mortality

## Abstract

**Background:**

The concept of a weekend effect, poorer outcomes for patients admitted to hospitals at the weekend is not new, but is the focus of debate in England. Many studies have been published which consider outcomes for patients on admitted at the weekend. This systematic review and meta-analysis aims to estimate the effect of weekend admission on mortality in UK hospitals.

**Methods:**

This is a systematic review and meta-analysis of published studies on the weekend effect in UK hospitals. We used EMBASE, MEDLINE, HMIC, Cochrane, Web of Science and Scopus to search for relevant papers. We included systematic reviews, randomised controlled trials and observational studies) on patients admitted to hospital in the UK and published after 2001. Our outcome was death; studies reporting mortality were included. Reviewers identified studies, extracted data and assessed the quality of the evidence, independently and in duplicate. Discrepancy in assessment was considered by a third reviewer. All meta-analyses were performed using a random-effects meta-regression to incorporate the heterogeneity into the weighting.

**Results:**

Forty five articles were included in the qualitative synthesis. 53% of the articles concluded that outcomes for patients either undergoing surgery or admitted at the weekend were worse. We included 39 in the meta-analysis which contributed 57 separate analyses. We found an effect of 1.07 [odds ratio (OR)] (95%CI:1.03–1.12), suggesting that patients admitted at the weekend had higher odds of mortality than those admitted during the week. Sub-group analyses suggest that the weekend effect remained when measures of case mix severity were included in the models (OR:1.06 95%CI:1.02–1.10), but that the weekend effect was not significant when clinical registry data was used (OR:1.03 95%CI: 0.98–1.09). Heterogeneity was high, which may affect generalisability.

**Conclusions:**

Despite high levels of heterogeneity, we found evidence of a weekend effect in the UK, even after accounting for severity of disease. Further work is required to examine other potential explanations for the “weekend effect” such as staffing levels and other organisational factors.

**Trial registration:**

PROSPERO International Prospective Register of Systematic Reviews -registration number: CRD42016041225.

**Electronic supplementary material:**

The online version of this article (10.1186/s12913-018-3688-3) contains supplementary material, which is available to authorized users.

## Background

The concept of a “weekend effect” is not new. From as early as the 1970s, researchers have reported poorer outcomes for patients admitted or treated at the weekends across a variety of medical settings, diagnoses, procedures and countries [1–4]. However, recently the “weekend effect” has prompted controversy, particularly in the UK (United Kingdom), as demonstrated by the increasing use of the common prefix in research papers and newspaper articles: ‘so-called’ [5, 6]. This controversy, particularly in the UK, appears to have been inflamed by an announcement on 13th October 2015 by the Secretary of State for Health, claiming that avoidable deaths occurred at weekends because there was not a full seven-day NHS (National Health Service) service; specifically he claimed that *“there are 11,000 excess deaths as a result of inadequate cover at weekends”* [7]. The BMJ subsequently pointed out that the research he cited to support his statement did not attribute the deaths to poor staffing and did not claim that the deaths were necessarily avoidable. The Secretary of State’s claim soon became part of an ongoing industrial dispute between junior doctors and the government about the introduction of a new contract. The dispute originally began in October 2013 and led to strike ballots in November 2015 [8]. The weekend effect became part of a public debate between junior doctor campaigners, academics and the Department of Health; research articles gained increased exposure and became a focus for journalists, health bloggers and clinicians.

Amidst the controversy a number of explanations have been put forward for the weekend effect. The first is that patients admitted at the weekend are ‘sicker’ and outcomes can therefore be expected to be worse. A second is that staffing levels are lower at weekends and this causes delays in diagnostics and procedures. A third explanation is that there is no weekend effect, and that outcomes for patients admitted at weekends are not worse and that studies who report this are actually seeing a statistical artefact. A number of systematic reviews have been published which might have assisted in settling the controversy. In 2014, Sorita et al. carried out two meta-analyses reporting the ‘off-hours effect’ for acute myocardial infarction and acute ischaemic stroke [9, 10]. Significant off-hours effects were found for both diagnoses. In a meta-analysis Zhou et al. [11] found an ‘off-hours effect’ for 20 out of 28 diseases, including several malignancies, cardiovascular disease and stroke. More recently, Pauls et al. [12] have published a meta-analysis of the “weekend effect”, and attempted to determine whether staffing is associated with the weekend effect. They found that patients admitted on the weekends had a significantly higher overall mortality. When analysing a subset of papers that included information on staffing, they found a significantly higher mortality for weekend patients, associated with decreased staffing levels, and no significant difference in mortality for weekend patients when staffing was similar to that for the weekdays. Hoshijima et al. [13] analysed the 88 international studies and found a 12% increased odds for short term mortality for patients admitted at the weekend and found a consistent effect across all continents. In line with Zhou et al. [11] they found a weekend effect in specific disease groups and suggest that this was related to these diseases needing urgent diagnosis and treatment. However, some disease groups had small numbers of studies (one or two). These systematic reviews were international in scope, which gives an important global picture of health care. Given the heterogeneity of healthcare systems internationally, and the UK centric focus of some controversy around the evidence for the weekend effect, we have systematically reviewed the evidence for the weekend effect on mortality within the public healthcare system (the National Health Service) solely in the UK. We also hypothesised that the date of publication (before and after the announcement by the Secretary of State’s claims surrounding the weekend effect), sample size, the data source and the extent of severity adjustment might impact upon the strength of the association between weekend admissions and mortality.

Our systematic review includes studies on patients admitted to hospital in the UK, either as elective or emergency patients, and published after 2001. In this review we confine the outcome to death defined by day of the week or combined as weekend/weekday.

The many reasons given to explain the weekend effect and explain different results led to us developing key questions to be investigated using sub-group analyses.

A) Are studies which find no weekend effect small and under-powered to detect a weekend effect?
B) Is the weekend effect only a result of more severe patients being admitted at the weekend?
  - Is a weekend effect found when clinical sets only are analysed, in comparison to administrative datasets which generally have more limited information on illness severity?
  - Does the weekend effect remain when studies which have highlighted severity measures in their analysis are included as a sub-group?

In addition, we hypothesised that studies published after the controversial statement by the Minister of Health that excess deaths were directly attributable to a weekend effect might bias researchers’ interpretations of results because of the perceived views of policy makers and appropriation of research results in support of an ongoing industrial dispute.

## Methods

### Search methods for identification of studies

The review protocol has been registered in the PROSPERO International Prospective Register of Systematic Reviews (registration number: CRD42016041225). The protocol considers both processes and outcomes: here we focus on outcomes. We hope to publish further work on processes in the future. The review has been written according to the Preferred Reporting Items for Systematic Reviews and Meta-Analyses (PRISMA) statement.

A primary search was carried out in July 2016. Studies were identified through 6 databases - EMBASE, MEDLINE, HMIC, Cochrane, Web of Science and Scopus. The full search strategy for each database is shown in Additional file 1. Further studies were identified from investigating study references, and a final search of MEDLINE was carried out in July 2017.

### Assessment of literature for inclusion

Two reviewers independently assessed the literature for inclusion in both the primary (A and B) and final (C and B) search.

### Inclusion criteria

For our systematic review, we included published systematic reviews, randomised controlled trials and observational studies. We excluded studies published prior to 2002 to reflect a period of relative stability in the provision of health care in the UK. We included studies on patients admitted to hospital in the UK regardless of age, admission type (elective or emergency), medical specialty or diagnosis at admission. Our comparison was weekend vs weekday and we included all studies that defined outcomes by day of the week or combined as weekend/weekday. Our outcome was death and only studies reporting mortality (in-hospital or all mortality over any time period up to one year) were included.

### Data extraction and quality assessment

Data was extracted by two reviewers: (A and B in the first search; C and B in second). Extracted data included date of study publication (where possible this was supplemented by date of submission), data source (clinical registry or administrative), comparison type (day of the week, or weekend vs weekday), day of baseline, admission type and adjustment approach. In addition, extracted data included sample size and estimate type (relative risk, odds ratio and hazard ratio).

This review used adapted CASP tools for evidence appraisal and bias assessment. [Casp2013] Utilising a series of questions, the CASP checklist assessed the study validity, application to research question, result significance and generalisability. The assessment of the bias was carried out separately by two reviewers (A and C) any discrepancy in assessment was considered by a third reviewer (B) to gain a consensus. Eight aspects of quality were reviewed: these are summarised in Table 1. We considered whether patients had been excluded without a clear rationale and whether the study described confounders and took these into account. Studies were considered generalizable if they covered a wider geographical area than a single hospital trust. The Kappa statistic was used to determine the inter-rater reliability prior to the third reviewer resolving discrepancies in assessment.

**Table 1:** Aspects of quality reviewed

1. Did the review answer a clearly focused question? 2. Was the cohort recruited in an acceptable way? 3. Was the exposure accurately measured to reduce bias? 4. Has the study identified any confounding factors? 5. Has the study taken into account all of the confounding factors in the analysis 6. Was the outcome accurately measured to minimise bias? 7. How precise are the results? 8. How generalizable are the results?	

### Data synthesis and analysis

Initially we summarised studies based on the extraction variables (e.g. publication and submission date, data source, and severity adjustment). In addition, all papers were read by two reviewers to determine the overall conclusion of the paper. The overall conclusion of the study was compared with results presented in the main findings of the paper. For each study, we determined whether a measure of severity was included in the case-mix adjustment. In order for an adjustment measure to be defined as ‘severity’ it had to be a clinical factor specific to the disease of focus in the study, or, if all cause, specific to the diagnosis of the individual patient. We did not consider comorbidities or medical history to be measures of severity.

In order to carry out the meta-analysis we included only studies with underlying data and not just effect estimates. We used the authors’ definition of weekend and mortality as the main outcome. When papers reported mortality over different time periods we selected 30-day as the main one. Adjusted outcomes were used in preference to unadjusted outcomes when both were given. In cases where there were multiple analyses for different diagnoses and a combined analysis, the estimates from the combined analysis (based on all diagnoses) were taken; when there was no combined analysis, the estimates for the individual diagnoses were taken. Studies reporting odds ratios and their confidence interval were included in the meta-analysis. Risk ratios were converted to odds ratios in studies where provided data allowed. Analyses estimating hazard ratios were not included in the main meta-analysis, but analysed separately.

To estimate heterogeneity we used the Cochran Q score (reported as I^2^). All meta-analyses were performed using a random-effects meta-regression to incorporate the heterogeneity into the weighting, which includes a measure of the study size.

We carried out subgroup analyses to investigate the effect of study factors to the overall effect estimate. We carried out four subgroup analyses: a) studies published before and after 13th October 2015 (the date of the Ministerial statement on the weekend effect); b) data sourced from clinical registries compared with data sourced from administrative sources; c) study sample size – divided into four categories determined a priori; and d) inclusion of severity measure in the adjustment approach compared with no inclusion of severity of measure.

### Patient involvement

We involved no patients in the development of the research question or in the selection of study design and outcome measures. No patients were involved in the conduct of the study. We do not plan to disseminate the results to study participants.

## Results

### Studies included

**Fig. 1:**
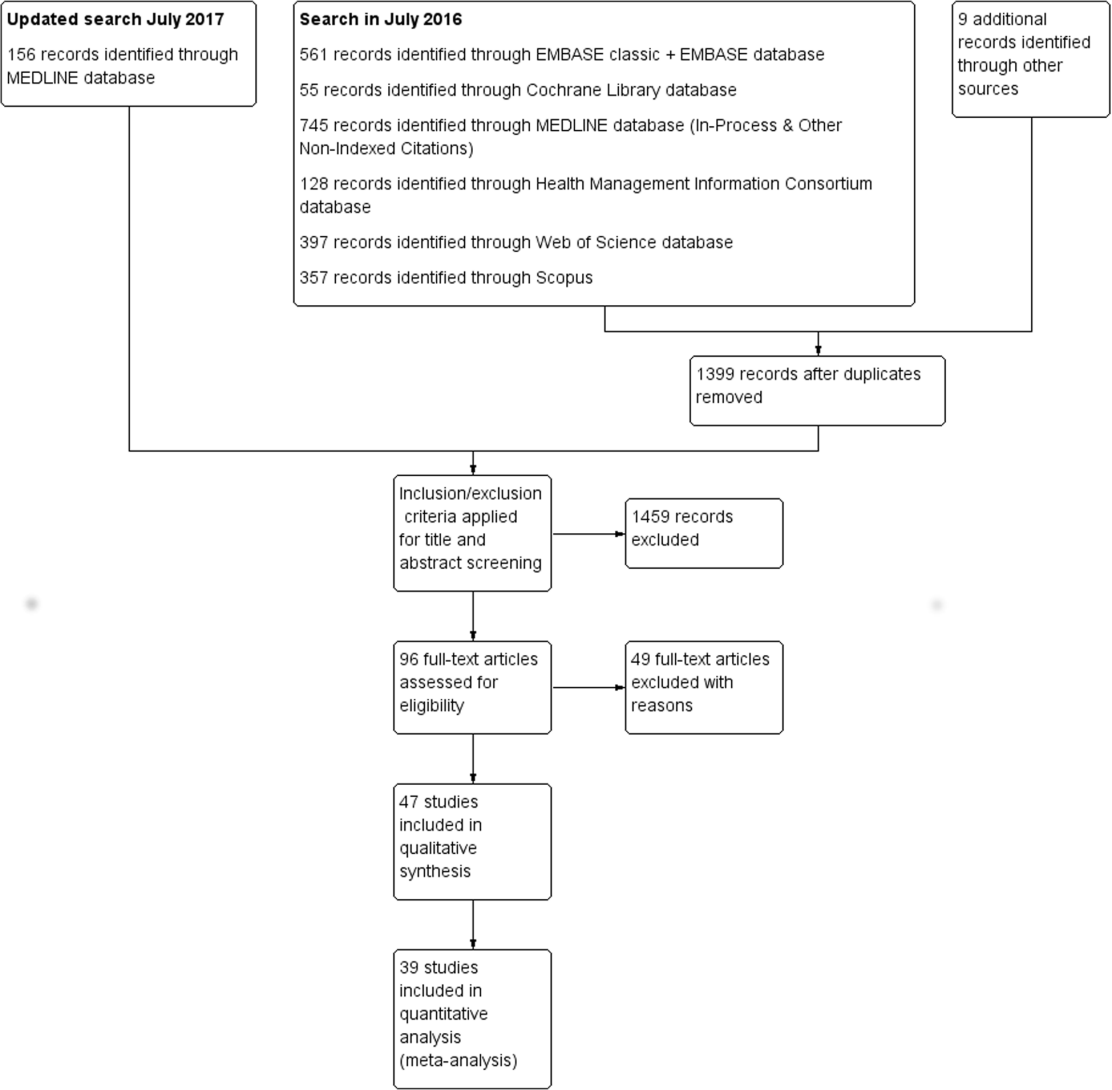
Flow diagram of studies selected for meta-analysis

We screened the title and abstract of 1555 articles and 96 were assessed for eligibility. 47 full-text articles initially met our inclusion criteria. All articles were observational studies. Two of these articles were later excluded from both the qualitative synthesis and quantitative analysis. Although McShane et al. [14] met the inclusion criteria, when we attempted to include it in the quantitative synthesis it was determined that it was not possible to separate the results for the UK and Ireland. In addition, Mohammed et al. [15] [retracted 2017] met the inclusion criteria but was later retracted. 45 articles were included in the qualitative synthesis and 39 in the quantitative analysis (see Fig. 1). Articles were excluded from the quantitative analysis when there was insufficient information reported, for example when the article reported statistics without information on the variability of the estimate. A summary of study characteristics and assessment of bias is included in the Additional file 1.

### Quality of studies

In general the quality of the papers was deemed to be good. They used validated data sources including clinical audits and administrative data sets and used recognised statistical methods to answer focussed questions. The majority of the studies adjusted for confounders within the scope of the data. The main bias identified in review is that clinical audits, by their nature, are quite small and this means confidence intervals are quite large affecting the precision of results and, although not evidence by our interpretation of the CASP question, different studies adjusted for different confounders, and this affects the robustness of the meta-analysis. Studies which focussed on one hospital trust or site were considered to have limited generalisability and this was another source of bias.

Agreement between 1st and 2nd reviewer ranged between 55 and 98% median 81%. All disputes were settled by reviewer 3. However, because of the large number of studies determined as good there was little variability resulting in low kappa statistics [range 0–0.66]. A lack of agreement centred on reviewers’ specific interpretation of certain CASP questions.

### Outcome

Although we extracted only papers that considered the outcome ‘death’, there was a wide variety in the specific way in which mortality was defined. 21 of the studies (47%) focussed on deaths in hospital, whereas 19 (42%) tracked patients after discharge and death in and out of hospital was the outcome. For five studies we were unable to determine whether patients were tracked after discharge. The most common time frame for tracking patients after death was 30 days, although this varied from one week to one year. The weekend was defined as midnight on Friday to midnight on Sunday in 21 studies, with the majority of other studies using the phrase Saturday and Sunday with no further detail given. One study used 16:00 on Friday to 16:00 on Sunday; [16] Saturday 07:00 to Monday 07:00 and Saturday 08:00 to Monday 08:00 were used by [17] et al. and [18] et al. respectively.

### Qualitative synthesis

#### Year of publication

There has been an increase in publication rate since 2002, with a steep increase in 2015 and 2016. We were particularly interested in the impact of the Secretary of State for Health’s claim of 11,000 excess deaths based on the Freemantle paper [19]. Therefore we also looked at the precise date of publication and the date of submission. Of the nine papers published in 2015, three papers were published after Oct 13 2015, and two of these were originally submitted before this date. It was not possible to determine the submission date of the third. Of the papers published in 2016 three papers were submitted before the date Secretary of State for Health’s statement. For four papers it was not possible to determine the date of submission.

#### Data sources

Only 11 studies had sample sizes of 100,000 or more; 10 studies had sample sizes of less than 1000. 28 studies analysed administrative data, NHS Hospital Episode Statistics or equivalent data sets from Scotland and Wales. Four studies used national stoke audit data, five other national audits were used and 10 local audits, which covered a range of diagnoses. The majority of studies (76%) included data from more than one year. The majority of studies (62%) were national (England, Scotland, Wales, Northern Ireland or some combination of these), and a further 20% were single-site studies. Other studies were either regional or a sample of sites from the UK.

#### Patient groups - diagnoses

A key difference in studies is whether they investigated all-cause outcomes or condition-specific outcomes. 14 studies focussed on all-cause admissions, although some of these analyse specific conditions within the papers. Eight studies consider stroke admissions only. Other specific conditions studied included upper gastrointestinal bleeding and COPD.The availability of clinical registries or audits affects is also related to whether specific diseases were studied.

#### Adjustment of outcomes and inclusion of severity measures

The majority analyses attempted to take into account potential confounders and severity at admission or attendance, but the approaches varied. We classified severity as being specific to the disease of focus, if applicable. We did not include proxies such as mode of arrival or referral, disease type or comorbidities as measures of severity. Fourteen studies included a measure of severity. One study considered all-cause emergency admissions, and the remaining studies were disease-specific (stroke:5; ICU:2; hip fracture:2; COPD:1; UGIB:1; and paediatric outcomes:1). Measures of severity varied between diseases. For example, both studies focussing on the outcomes of hip fracture patients used the American Society of Anaesthesiologists physical status classification. Both ICU studies used the APACHE II system, although one study used the combined score and one used individual components. The stroke studies used a variety of measures of severity including the National Institutes of Health Stroke Score, the modified Rankin scale, the worst level of consciousness in the previous 24 h and whether a palliative care decision was made in the first 24 h.

In addition to studies that included a measure of severity, various studies claimed to use proxies for severity, including mode of arrival at hospital, arguing that arriving by ambulance was a proxy for more severe patients [20]. Several studies categorise patients based on the clinical risk associated with the primary diagnoses.

#### Narrative analysis

All abstract conclusions were read by two reviewers. We determined whether the emphasis of the conclusion was that there was no weekend effect, a weekend effect or no mention of the effect. Three studies did not mention the weekend effect as part of their conclusions. For two of these, day of the week was not part of the aim set out in the abstract. A third study did mention the higher weekend mortality in the abstract, but this was not part of a specific aim. For 24 of the studies the abstract concluded that outcomes for patients either undergoing surgery or admitted at the weekend were worse. The proportion of studies reporting a weekend effect was higher before the Secretary of State’s statement (65% compared with 37%). 18 studies concluded that there was no effect, 31% of those published before and 53% of those published after the controversial statement by the Secretary of State. Of the studies which reported no effect [5, 20–34] there was some evidence that the results of the statistical analysis indicated worse outcomes for patients admitted at weekends (two of the eight published before [21, 33] and six of the 10 published after [20, 24, 28, 31, 33, 35]). These included studies that tried different methods of adjustment, for example Wunsch et al. [22] who tried two forms of adjustment, one which resulted in a significant effect and which did not, and used the phrase “After appropriate adjustment”. Anselmi et al. [20] also used different adjustment methods, and found that “Using conventional risk-adjustment methods, there appears to be a higher risk of mortality …..”. When model of arrival was included in the adjustment approach there was no significant effect. One study aimed to mitigate any effect by using the phrase ‘limited effect’ [35].

### Quantitative analysis

**Fig. 2:**
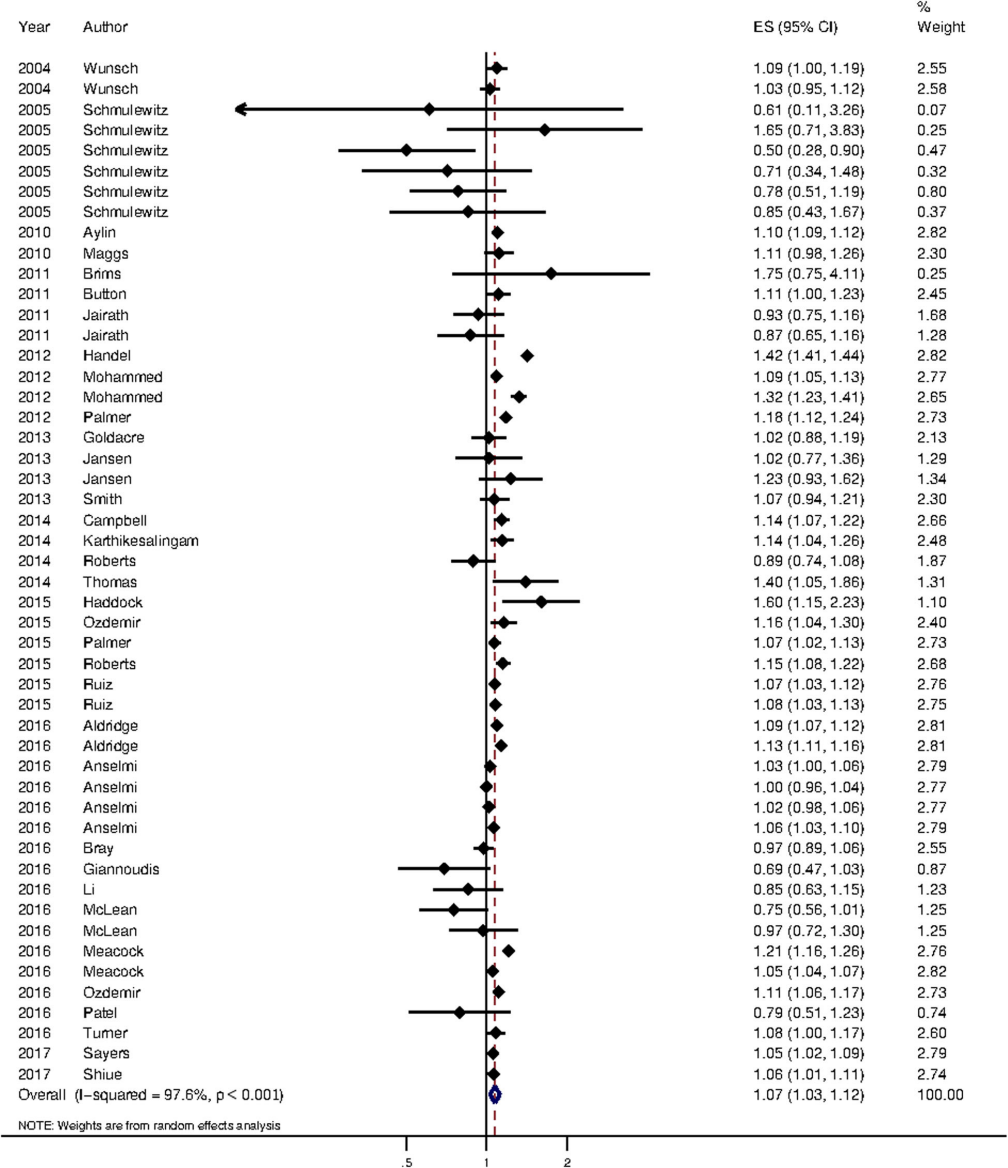
Pooled estimate for mortality between weekend and weekday patients. Patients who were admitted at the weekends had a significantly higher mortality (OR: 1.07; 95% CI 1.03 to 1.12)

34 articles were included in the main meta-analysis, which contributed 50 separate analyses. These are summarised in Table 2. Meta-analysis on these studies showed that patients admitted at the weekend had a significantly higher mortality than those admitted during the week (OR = 1.07, 95% CI: 1.03 to 1.12). These are summarised in Fig. 2. Five articles, reporting hazard ratios in 7 analyses, included in a separate meta-analysis, had similar findings (HR = 1.09, 95% CI: 1.05 to 1.14), see Additional file 2.

**Table 2:** Summary of studies included in the main meta-analysis

Study	Year of publication	No. of analyses included in meta-analysis	Date of publication	Database type	Study sample size	Measure of severity
Aldridge et al. [41]	2016	2	Post-13/10/15	Administrative	Over 1,000,000	No measure of severity
Anselmi et al. [20]	2016	4	Post-13/10/15	Administrative	Over 1,000,000	No measure of severity
Aylin et al. [2]	2010	1	Pre-13/10/15	Administrative	Over 1,000,000	No measure of severity
Bray et al. [5]	2016	1	Post-13/10/15	Clinical registry/audit	10,000 to 100,000	Measure of severity
Brims et al. [42]	2011	1	Pre-13/10/15	Administrative	Less than 10,000	Measure of severity
Button et al. [43]	2011	1	Pre-13/10/15	Administrative	10,000 to 100,000	No measure of severity
Campbell et al. [34]	2014	1	Pre-13/10/15	Clinical registry/audit	10,000 to 100,000	Measure of severity
Giannoudis et al. [39]	2016	1	Post-13/10/15	Clinical registry/audit	Less than 10,000	No measure of severity
Goldacre et al. [25]	2013	1	Pre-13/10/15	Administrative	10,000 to 100,000	No measure of severity
Haddock et al. [28]	2015	1	Post-13/10/15	Administrative	Less than 10,000	No measure of severity
Handel et al. [44]	2012	1	Pre-13/10/15	Administrative	Over 1,000,000	No measure of severity
Jairath et al. [21]	2011	2	Pre-13/10/15	Clinical registry/audit	Less than 10,000	Measure of severity
Jansen et al. [23]	2013	2	Pre-13/10/15	Administrative	Less than 10,000	Measure of severity
Karthikesalingam et al. [45]	2014	1	Pre-13/10/15	Administrative	10,000 to 100,000	No measure of severity
Li et al. [29]	2016	1	Post-13/10/15	Clinical registry/audit	10,000 to 100,000	Measure of severity
Maggs et al. [46]	2010	1	Pre-13/10/15	Administrative	10,000 to 100,000	No measure of severity
McLean et al. [47]	2016	2	Post-13/10/15	Administrative	100,000 to 1,000,000	No measure of severity
Meacock et al. [24]	2017	2	Post-13/10/15	Administrative	100,000 to 1,000,000	No measure of severity
Mohammed et al. [48]	2012	2	Pre-13/10/15	Administrative	Over 1,000,000	No measure of severity
Ozdemir et al. [49]	2015	1	Pre-13/10/15	Administrative	Less than 10,000	No measure of severity
Ozdemir et al. [50]	2016	1	Post-13/10/15	Administrative	100,000 to 1,000,000	No measure of severity
Palmer et al. [51]	2012	1	Pre-13/10/15	Administrative	10,000 to 100,000	No measure of severity
Palmer et al. [18]	2015	1	Post-13/10/15	Administrative	Over 1,000,000	No measure of severity
Patel et al. [31]	2016	1	Post-13/10/15	Administrative	10,000 to 100,000	No measure of severity
Roberts et al. [27]	2014	1	Pre-13/10/15	Administrative	10,000 to 100,000	No measure of severity
Roberts et al. [52]	2015	1	Pre-13/10/15	Administrative	10,000 to 100,000	No measure of severity
Ruiz et al. [17]	2015	2	Pre-13/10/15	Administrative	100,000 to 1,000,000	No measure of severity
Sayers et al. [35]	2017	1	Post-13/10/15	Clinical registry/audit	100,000 to 1,000,000	Measure of severity
Schmulewitz et al. [26]	2005	6	Pre-13/10/15	Administrative	Less than 10,000	No measure of severity
Shiue et al. [32]	2017	1	Post-13/10/15	Administrative	100,000 to 1,000,000	No measure of severity
Smith et al. [30]	2014	1	Pre-13/10/15	Administrative	10,000 to 100,000	No measure of severity
Thomas et al. [53]	2014	1	Pre-13/10/15	Administrative	Less than 10,000	Measure of severity
Turner et al. [54]	2016	1	Post-13/10/15	Administrative	10,000 to 100,000	Measure of severity
Wunsch et al. [22]	2004	2	Pre-13/10/15	Clinical registry/audit	10,000 to 100,000	Measure of severity

#### Subgroup analyses

Table 3 shows the results of the sub-group analyses. There was no evidence that there was any association between publication date and weekend outcomes. Regardless of population size, there is evidence of a higher mortality rate for weekend admissions. However, the confidence interval for the studies with the smallest populations includes one, which suggests that when only small studies are analysed there is limited evidence to support the weekend effect. Studies based on clinical registry/audit data did not show that weekend patients had significantly worse outcomes. However, none of the studies with 100,000 patients or more were clinical registries/audits. The division between administrative and clinical data sets is often used a proxy for having clinical information. We also divided studies into those that included measures of severity and those that did not. We did not find an association between studies which included a measure of severity and higher mortality for weekend admissions.

**Table 3:** Sub-group analyses

Subgroup	Number of analyses	OR (95% CI)	*p*-value	I^2^ (%)
All Analyses^a^	50	1.07 (1.03, 1.12)	0.002	97.6
Date of publication^b^				
• Pre-October 13th 2015^c^	30	1.09 (1.02, 1.17)	0.009	97.5%
• Post-October 13th 2015	20	1.06 (1.03, 1.09)	< 0.001	84.0%
Study sample size				
• Less than 10,000	15	1.03 (0.89, 1.18)	0.72	57.9%
• 10,000 to 100,000	15	1.08 (1.04, 1.13)	< 0.001	57.1%
• 100,000 to 1,000,000	8	1.08 (1.04, 1.30)	0.001	81.9%
• Over 1,000,000	12	1.11 (1.02, 1.21)	0.015	99.4%
Database type				
• Administrative	41	1.09 (1.04, 1.15)	< 0.001	98.0%
• Clinical registry/audit	9	1.03 (0.98, 1.09)	0.25	57.8%
Measure of severity				
• No measure of severity	37	1.08 (1.03, 1.14)	0.004	98.2%
• Measure of severity	13	1.06 (1.02, 1.10)	< 0.001	43.5%

^a^50 analyses from 34 published articles

^b^Date of publication was used rather than submission as date of submission was not available for all articles

^c^The 13th October 2015 was the date of the Minister Of Health’s statement in the House of Commons about the weekend effect

## Discussion

### Main findings

We found evidence that when studies are combined there is evidence of a weekend effect, with patients admitted at the weekend having higher odds of mortality that was not explained by measures of severity included in the studies. However, there were high levels of heterogeneity in the meta-analysis, suggesting there may be some concerns about the generalisability of the result. We found considerable variation in the approaches taken, including the time period for mortality, the definition of weekend and the variables included as measures of severity, which all contribute to the high levels of heterogeneity and are likely to be a cause of some of the conflicting results and their interpretation.

We included 45 papers in the qualitative analysis. The majority of these were published in the last three years. Just over half of these studies (53%) concluded that outcomes for patients either undergoing surgery or admitted at the weekend were worse. The proportion of papers that concluded that outcomes for weekend patients were worse decreased after the statement by the Secretary of State in October 2015. We included 34 papers in the meta-analysis which contributed 50 studies, 26 of which found evidence of a weekend effect. We found an overall effect of 1.07 [odds ratio (OR)] (95% CI 1.03–1.12), suggesting that patients admitted at the weekend had higher odds of mortality that those admitted during the week. Sub-group analyses suggest that the weekend effect remained when measures of severity were included in the models, but that the weekend effect was not significant when clinical registry data was used.

### Strengths and weaknesses

This study is the first review to focus on admissions to hospitals in the United Kingdom (UK). This is a strength in that hospitals across the UK are relatively homogenous and although patient demographics vary, the health system is comparable across regions of the UK. The main limitation of the study is the high heterogeneity, which means the estimate of the effect size from the meta-analysis may not be valid. The high heterogeneity was expected as the nature of the studies we included varied in terms of size, disease, time to outcome and other factors and is in line with other systematic reviews on the weekend effect [12]. Some of the sub-groups exhibited lower heterogeneity and the weekend effect remained significant, for example studies which included a measure of severity. The restriction of studies to those within the UK limits the generalisability of the findings to other countries but is important for local and national policy, and other studies have already established that the weekend effect is an international phenomenon. We consider the categorisation of papers into those that include a severity measure to be a key strength as a common explanation of the weekend effect is that more severe patients are admitted at the weekend (for example see [24]). Previous systematic reviews [11, 13] carried out sub-group analyses on different diagnostic categories, we were concerned that the small numbers of studies in the majority of categories would not add this area. Our investigation of the impact of a major political announcement on research publications is important. It has been shown that confirmation bias can affect how researchers interpret results [36] and unconscious bias can influence research evaluation [37]. However, we acknowledge that we have only completed an initial analysis, and there is the risk of our own bias influencing the interpretation of abstracts. There is also the risk of bias as two of the authors (D and B) of this paper are also authors of various research papers and commentaries on the weekend effect. We endeavoured to overcome this potential bias by involving researchers (E, C and A) who have not previously published on this topic and were new to much of the literature. We did not attempt to determine whether the outcomes were associated with quality of care or weekend staffing, and we cannot offer explanations as to the cause of the weekend effect.

This review has shown that hospital mortality does differ between weekends and weekdays in the UK, consistent with two recent reviews, both showing poorer outcomes for patients admitted at the weekend [11, 12]. Despite the increase in publications in recent years and the rise in discussion of the topic in the media, we did not find an association between the date of publication and the relative risk of mortality when we carried out a sub-group meta-analysis. However, our narrative analysis, which considered the overall conclusions drawn by the authors, showed evidence of change over time. The increasing need for health services research to have impact has led to researchers increasingly choosing to study topics with policy leverage and ‘present them in a manner that policy makers think about these issues’ [38]. These pressures may influence not only topics for research, but also interpretation and publication. Sub-group analyses showed that regardless of sample size, there was a higher risk of worse outcomes for patients admitted at the weekend, but that this was not significant for smaller sample sizes. This may be due to a lack of power to detect significant differences in smaller samples, or that smaller studies may use different data sources or come from sites with different case-mix or with different weekend care.

We did find that the data source was associated with the weekend effect, with a non-significant effect for studies based on clinical data. However, when we divided studies based on the inclusion of a severity measure the weekend effect remained in both groups. We found that the use of clinical audit data did not necessarily mean that measures of severity were included in the analysis [39] and that measures of severity inevitably varied. In a systematic review of the effect of weekend admission on outcomes for patients with upper gastrointestinal bleeding, variceal bleeding was not associated with weekend admission, but non-variceal bleeding was, suggesting that a more sophisticated approach than a ‘severity measure’ may be important and may be disease-specific [40].

## Conclusion

In this systematic review, we found evidence of a weekend effect. However, the high levels of heterogeneity in study design, including outcomes and the inclusion of confounder, mean it is not possible to quantify the effect accurately. We suggest that individual hospital managers examine their own performance carefully and if poorer outcomes are found for weekend admissions possible reasons are examined. We found a weekend effect even after accounting for severity of disease, further work is required to examine other potential explanations for the “weekend effect” such as staffing levels and other organisational factors.

## Additional files

Additional file 1: Free Text and MeSH Heading terms used in Literature Search. Baseline characteristics of all studies. Assessment of bias using CASP questions. (DOCX 159 kb)

Additional file 2: **Figure 1.** Flow diagram of studies selected for meta-analysis. **Figure 2.** Additional meta-analysis of studies reporting hazard ratios. **Table 1.** Summary of studies in additional meta-analysis (those reporting hazard ratios). (DOCX 172 kb)

## Abbreviations

CASP: Critical Appraisal Skills Programme
CI: Confidence interval
COPD: Chronic obstructive pulmonary disorder
I^2^: Measure of heterogeneity
ICU: Intensive care unit
NHS: National Health Service
OR: Odds ratio
UGIB: Upper gastro-intestinal bleeding
UK: United Kingdom
