# Supplementary material for: The weekend effect: does hospital mortality differ by day of the week? A systematic review and meta-analysis
Source: BMC Health Serv Res. 2018 Nov 20;18:870. doi: 10.1186/s12913-018-3688-3 (PMC6245775; doi:10.1186/s12913-018-3688-3)
Supplement: Supplementary file 1 — Free Text and MeSH Heading terms used in Literature Search. Baseline characteristics of all studies. Assessment of bias using CASP questions. (DOCX 159 kb) [file 12913_2018_3688_MOESM1_ESM.docx]

**Appendices**

1. **Free Text and MeSH Heading terms used in Literature Search**

Relevant literature was searched through 6 electronic databases. They included the following: EMBASE Classic + EMBASE (1947 to 2016 July 06), MEDLINE In-Process & Other Non-Indexed Citations (1946 to present), the Health Management Information Consortium (1979 to May 2016), Cochrane Library (all years), Web of Science (all years) and Scopus (all years to present). There were no restrictions on language nor publication period. The search was conducted in early July 2016.

**EMBASE**

Embase Classic+Embase 1947 to 2016 July 06

Date search was undertaken: 6^th^ July 2016

Person who ran the search: Lo Ka Man Michelle

Portal: OVID

No. of hits: n= 561

1. (monday* or tuesday* or wednesday* or thursday* or friday* or saturday* or sunday* or weekend* or week-end* or (week adj3 end) or (day* adj3 week*) or weekday* or week-day* or holiday* or out-of-hour* or after-hour* or working-week* or working-day*).mp. [mp=title, abstract, heading word, drug trade name, original title, device manufacturer, drug manufacturer, device trade name, keyword]
2. exp working time/
3. 1 or 2
4. ((hospital* adj3 outcome*) or (patient* adj3 outcome*) or hospital-acquired-condition* or (Mortalit* or Death* or Survival* or Morbidit* or Complication*) or (quality adj2 care) or ((length* or duration* or time*) adj3 stay) or (Discharge adj3 patient*) or ((adverse or never or serious) adj1 event*) or (Error* or Mistake*) or (Patient* adj3 safety) or Rehospitali?ation* or Re-hospitali?ation* or Readmission* or Re-admission*).mp. [mp=title, abstract, heading word, drug trade name, original title, device manufacturer, drug manufacturer, device trade name, keyword]
5. exp mortality/ or outcome assessment/ or treatment outcome/ or patient safety/ or patient harm/ or "length of stay"/ or hospital readmission/ or exp morbidity/ or iatrogenic disease/ or exp medical error/ or hospital discharge/ or exp complication/ or exp diagnostic error/ or health care quality/
6. 4 or 5
7. ((process* adj2 care) or (patient* adj3 care) or ((availabilit* or timel* or delay* or omission* or (waiting adj2 time)) adj3 (diagnos* or treatment* or procedure* or care* or service* or investigati* or operat* or surg* or therap* or (patient* adj3 review*) or (patient* adj3 assess*)))).mp. [mp=title, abstract, heading word, drug trade name, original title, device manufacturer, drug manufacturer, device trade name, keyword]
8. exp patient care/
9. 7 or 8
10. 6 or 9
11. (emergency-admission* or (Emergen* adj2 admi*) or Elective-admission* or (Elective* adj2 admi*) or (Hospital adj3 admission*) or (Patient adj3 admission*) or Hospitali?ation* or (Hospital adj3 (treatment* or care or service*)) or Tertiary-care* or Secondary-care* or ((Inpatient or in-patient) adj (service* or care or treatment*))).mp. [mp=title, abstract, heading word, drug trade name, original title, device manufacturer, drug manufacturer, device trade name, keyword]
12. hospital admission/ or hospitalization/
13. 11 or 12
14. ((united adj kingdom) or UK or England or english or (Great adj Britain) or NHS or british or welsh or Wales or Scotland or scottish or (north* adj ireland) or (north* adj irish)).mp. [mp=title, abstract, heading word, drug trade name, original title, device manufacturer, drug manufacturer, device trade name, keyword]
15. exp United Kingdom/
16. 14 or 15
17. 3 and 10 and 13 and 16

**Medline**

Epub ahead of print, in-process and other non-indexed citations, OVID MEDLINE® Daily and OVID MEDLINE ®; 1946 to present

Date search was undertaken: 7^th^ July 2016

Person who ran the search: Lo Ka Man Michelle

Portal: OVID

No. of hits: n= 745

1. (monday* or tuesday* or wednesday* or thursday* or friday* or saturday* or sunday* or weekend* or week-end* or (week adj3 end) or (day* adj3 week*) or weekday* or week-day* or holiday* or out-of-hour* or after-hour* or working-week* or working-day*).mp. [mp=title, abstract, original title, name of substance word, subject heading word, keyword heading word, protocol supplementary concept word, rare disease supplementary concept word, unique identifier]

1. ((hospital* adj3 outcome*) or (patient* adj3 outcome*) or hospital-acquired-condition* or (Mortalit* or Death* or Survival* or Morbidit* or Complication*) or (quality adj2 care) or ((length* or duration* or time*) adj3 stay) or (Discharge adj3 patient*) or ((adverse or never or serious) adj1 event*) or (Error* or Mistake*) or (Patient* adj3 safety) or Rehospitali?ation* or Re-hospitali?ation* or Readmission* or Re-admission*).mp. [mp=title, abstract, original title, name of substance word, subject heading word, keyword heading word, protocol supplementary concept word, rare disease supplementary concept word, unique identifier]
2. "outcome assessment (health care)"/ or failure to rescue, health care/ or exp Mortality/ or "Quality of Health Care"/ or "length of stay"/ or patient discharge/ or patient readmission/ or Morbidity/ or Patient Safety/ or Pregnancy Complications, Hematologic/ or Postoperative Complications/ or Pregnancy Complications, Infectious/ or Pregnancy Complications, Parasitic/ or Obstetric Labor Complications/ or Pregnancy Complications, Neoplastic/ or Intraoperative Complications/ or Pregnancy Complications/ or Diabetes Complications/ or Pregnancy Complications, Cardiovascular/ or medical errors/ or diagnostic errors/ or medication errors/ or near miss, healthcare/ or Iatrogenic Disease/
3. ((process* adj2 care) or (patient* adj3 care) or ((availabilit* or timel* or delay* or omission* or (waiting adj2 time)) adj3 (diagnos* or treatment* or procedure* or care* or service* or investigati* or operat* or surg* or therap* or (patient* adj3 review*) or (patient* adj3 assess*)))).mp. [mp=title, abstract, original title, name of substance word, subject heading word, keyword heading word, protocol supplementary concept word, rare disease supplementary concept word, unique identifier]
4. exp Patient Care/ or "Process Assessment (Health Care)"/
5. 2 or 3 or 4 or 5
6. ((united adj kingdom) or UK or England or english or (Great adj Britain) or NHS or british or welsh or Wales or Scotland or scottish or (north* adj ireland) or (north* adj irish)).mp. [mp=title, abstract, original title, name of substance word, subject heading word, keyword heading word, protocol supplementary concept word, rare disease supplementary concept word, unique identifier]
7. exp Great Britain/
8. 7 or 8
9. (emergency-admission* or (Emergen* adj2 admi*) or Elective-admission* or (Elective* adj2 admi*) or (Hospital adj3 admission*) or (Patient adj3 admission*) or Hospitali?ation* or (Hospital adj3 (treatment* or care or service*)) or Tertiary-care* or Secondary-care* or in-hospital or inhospital or ((Inpatient or in-patient or in-hospital or inhospital) adj (service* or care or treatment*))).mp. [mp=title, abstract, original title, name of substance word, subject heading word, keyword heading word, protocol supplementary concept word, rare disease supplementary concept word, unique identifier]
10. exp Hospital Medicine/ or hospitalization/ or patient admission/
11. 10 or 11
12. 1 and 6 and 9 and 12

**2 Baseline characteristics of all studies**

| **Category** | **Number of research papers** |
| --- | --- |
| **Region** |  |
| - England | 32 |
| - Scotland | 7 |
| - Wales | 3 |
| - England and Wales | 2 |
| - England, Wales, Scotland and Northern Ireland | 1 |
| **Study duration** |  |
| - less than one year | 11 |
| - 1-5 years | 19 |
| - more than 5 years | 15 |
|  |  |
| **Study sample size** | 66 to 14 818 374 (total of approximately 60 million) |
| - Less than 1000 | 10 |
| - 1000 to 10, 000 | 7 |
| - 10,000 to 100, 000 | 17 |
| - 100,000 to 1, 000, 000 | 9 |
| - More than 1, 000, 000 | 2 |
| **Data sources** |  |
| - Hospital Episode Statistics (HES) | 22 studies (52611923) |
| - Patient Episode Database for Wales (PEDW) | 3 studies (72 898) |
| - Scottish Morbidity Database | 3 studies (5 352 042) |
| - National Hip Fracture Database | 2 studies (244 433) |
| - Stroke Audit Programmes | 4 national (228975) + 2 local (2758) local stroke audit |
| - Other audits (national) | 3 studies (64029) |
| - Other audits (local) | 8 studies (581249) |
| **Type of study** |  |
| - National | 28 |
| - Regional | 6 |
| - Single site | 10 |
| - Other (28 trusts across England) | 1 |
| Year of publication |  |
| - <2010 | 2 |
| - 2010 | 3 |
| - 2011 | 2 |
| - 2012 | 4 |
| - 2013 | 5 |
| - 2014 | 4 |
| - 2015 | 9  (6 published prior to 13/10/15)  (8 submitted prior to 13/10/15) |
| - 2016 | 13  (3 submitted prior to 13/10/15) |
| - 2017 | 3 |
| **Patient group – Diagnoses** |  |
| All cause emergency admissions | 10 |
| All admissions | 4 |
| Stroke (including sub-arachnoid haemorrhage) admissions | 8 |
| rAAA | 3 |
| UGIB | 3 |
| Hip | 2 |
| ICU | 2 |
| Other specific conditions | 8 |
| Other   - Emergency medical/general surgery - Selected emergency admissions - Acute medical admissions - Emergency admissions under a general surgeon (70+) | 2  1  1  1 |

**3. Assessment of bias using CASP questions.**
