# Supplementary material for: The weekend effect: does hospital mortality differ by day of the week? A systematic review and meta-analysis
Source: BMC Health Serv Res. 2018 Nov 20;18:870. doi: 10.1186/s12913-018-3688-3 (PMC6245775; doi:10.1186/s12913-018-3688-3)
Supplement: Supplementary file 2 — Figure 1. Flow diagram of studies selected for meta-analysis. Figure 2. Additional meta-analysis of studies reporting hazard ratios. Table 1. Summary of studies in additional meta-analysis (those reporting hazard ratios). (DOCX 172 kb) [file 12913_2018_3688_MOESM2_ESM.docx]

| Additional file 2 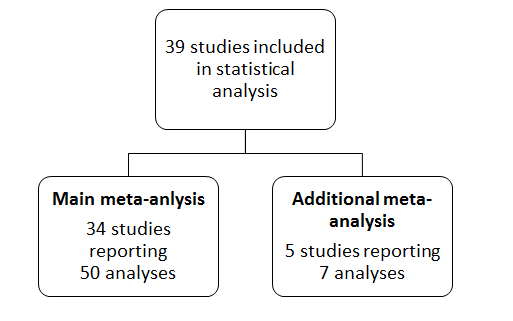  Additional file 2: Figure 1. Flow diagram of studies selected for meta-analysis | 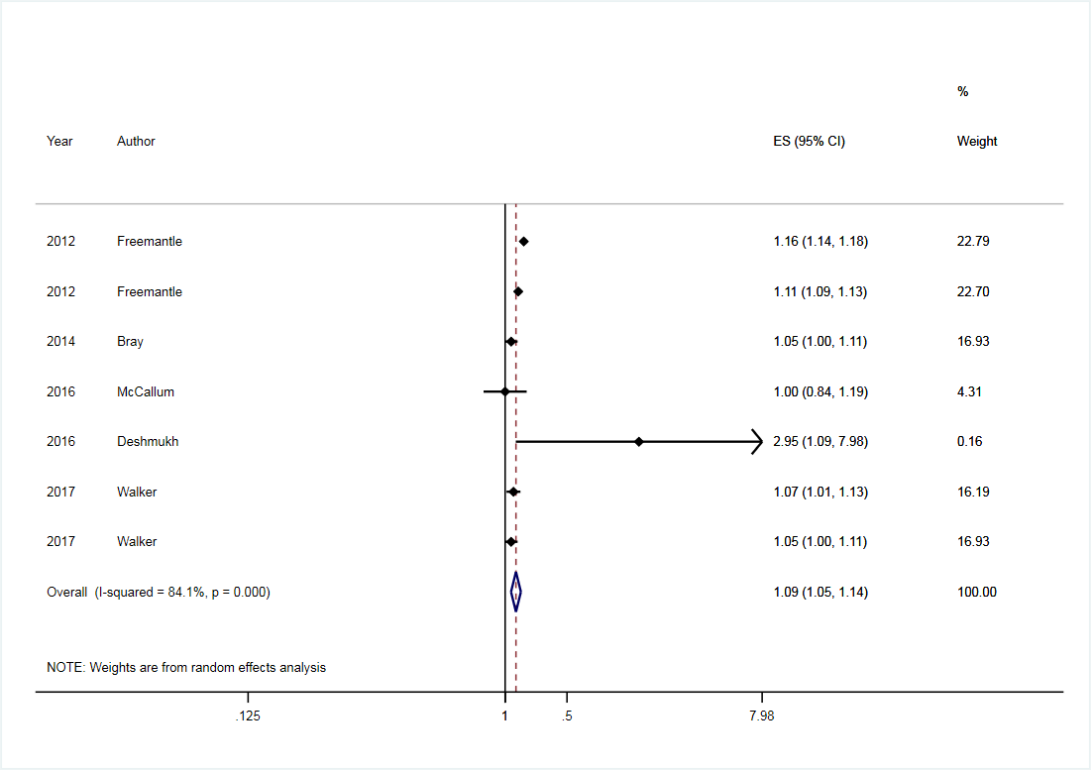 Additional file 2: Figure 2. Additional meta-analysis of studies reporting hazard ratios |
| --- | --- |

Additional file 2: Table 1. Summary of studies in additional meta-analysis (those reporting hazard ratios)

| Study | Year of publication | No of analyses included in meta-analysis | Date of publication | Database type | Study sample size | Measure of severity |
| --- | --- | --- | --- | --- | --- | --- |
| Freemantle et al. [19] | 2012 | 2 | Pre-13/10/15 | Administrative | Over 1,000,000 | No measure of severity |
| Bray [55] | 2014 | 1 | Pre-13/10/15 | Clinical registry/audit | 10,000 to 100,000 | Measure of severity |
| Deshmukh et al. [16] | 2016 | 1 | Post-13/10/15 | Clinical registry/audit | Less than 10,000 | Measure of severity |
| McCallum et al. [56] | 2016 | 1 | Post-13/10/15 | Administrative | 100,000 to 1,000,000 | No measure of severity |
| Walker et al. [33] | 2017 | 2 | Post-13/10/15 | Administrative | 100,000 to 1,000,000 | Measure of severity  (Blood test results) |

55. Bray BD, Ayis S, Campbell J, Cloud GC, James M, Hoffman A, et al. Associations between stroke mortality and weekend working by stroke specialist physicians and registered nurses: Prospective multicentre cohort study. PLoS Medicine. 2014;11(8):no pagination.

56. McCallum IJ, McLean RC, Dixon S, O'Loughlin P. Retrospective analysis of 30-day mortality for emergency general surgery admissions evaluating the weekend effect. The British journal of surgery. 2016;103(11):1557-65. doi:10.1002/bjs.10261.
